# Ovarian cancer with high-level focal *ERBB2* amplification responds to trastuzumab and pertuzumab

**DOI:** 10.1016/j.gore.2021.100787

**Published:** 2021-05-19

**Authors:** Laure Thouvenin, Mélinda Charrier, Sophie Clement, Yann Christinat, Jean-Christophe Tille, Mauro Frigeri, Krisztian Homicsko, Olivier Michielin, Alexandre Bodmer, Pierre O. Chappuis, Thomas A. McKee, Petros Tsantoulis

**Affiliations:** a Department of Oncology, University Hospitals of Geneva (HUG), Geneva, Switzerland; b Department of Genetic Medicine, Laboratory and Pathology, University Hospitals of Geneva (HUG), Geneva, Switzerland; c Multidisciplinary Oncology Center, Lausanne University Hospital (CHUV), Lausanne, Switzerland

**Keywords:** Ovarian cancer, ERBB2 amplification, Trastuzumab, Pertuzumab, Precision oncology

## Abstract

Epithelial ovarian cancer (EOC) is usually diagnosed at an advanced stage and significantly contributes to cancer mortality in women. Despite multimodal treatment associating chemotherapy and surgery, most patients ultimately progress and require palliative systemic therapy.

In EOC, the efficacy of anti-HER2 agents is minimal even after selecting patients for HER2 expression. *ERBB2* gene amplification is observed in 3–10% of patients, depending on the specific method of detection and cutoffs. We report the case of a young woman with a FIGO stage IV high-grade serous ovarian cancer with an amplification of *ERBB2*. She was treated with the association of trastuzumab – pertuzumab after two lines of standard treatment and presented an excellent long-lasting partial response after 36 months of treatment.

The association of trastuzumab and pertuzumab, without chemotherapy, has not been previously tested in this context and could be more efficacious than monotherapy with either agent. In addition, the significant benefit observed in this case could be attributed to the presence of a high-level focal amplification that is relatively rare and probably more specific than an increase in HER2 expression. In conclusion, prospective trials of the trastuzumab and pertuzumab combination should be considered in an appropriately selected EOC patient population.

## Highlights

• Trastuzumab monotherapy has limited efficacy in ovarian cancer.
• The combination of trastuzumab and pertuzumab has not been studied adequately.
• An ERBB2-amplified ovarian carcinoma had a durable response to this combination.
• High-level focal amplification may be a valuable biomarker predicting response.

## 1 Introduction

Ovarian cancer is the second most frequent gynecological cancer and the fifth most common cause of cancer death in women. High grade serous ovarian carcinoma (HGSOC) is the most frequent histological subtype and is associated with an aggressive behavior, so most cases are diagnosed at advanced stages. The standard of care of HGSOC involves surgical cytoreduction of all macroscopic disease followed by platinum-based adjuvant chemotherapy. Despite this multimodal approach, 70% of patients will relapse in the first three years (Ledermann et al., 2018). Systemic treatment options are limited after recurrence, although the advent of polyADP ribose polymerase inhibitors (PARPi) has led to longer progression free survival (PFS) (Franzese et al., 2019). The development of new therapies remains an unmet medical need.

Here, we report the case of a 46 year-old Caucasian woman diagnosed with advanced HGSOC with a focal amplification of the *ERBB2* gene who had a durable partial response to the association of pertuzumab and trastuzumab without concomitant chemotherapy. Trastuzumab is a monoclonal antibody which binds to the extracellular domain of the HER2 receptor. Pertuzumab is a monoclonal antibody which inhibits the dimerization of HER2 with other HER family receptors like HER3. Trastuzumab and pertuzumab have been used in combination for the treatment of breast cancer.

## 2 Case presentation

The patient presented with right abdominal pain in December 2016, motivating a CT-scan which showed peritoneal thickening, ascites, and a right pleural effusion. The CA-125 concentration was slightly elevated at 76 kU/l (upper limit of normal (ULN) < 35 kU/l), and laparoscopy revealed widespread peritoneal carcinomatosis and penetrating capsular liver lesions. The histological diagnosis showed a high grade epithelial serous carcinoma compatible with an ovarian origin.

Neoadjuvant treatment was administered with intravenous (IV) carboplatin (AUC 5, every 3 weeks) and paclitaxel (80 mg/m^2^, weekly). After three cycles, the CA-125 concentration decreased from 76 kU/l to 20 kU/l, and the CT scan showed a slight increase of the pleural infiltration, stability of the capsular liver lesion and peritoneal carcinomatosis without ascites. Cytoreductive surgery was performed in March 2017, allowing complete macroscopic resection of the tumor. Three additional cycles of adjuvant chemotherapy (carboplatin-paclitaxel) were administered, but the patient complained of right abdominal pain during the last cycle, and a CT-scan showed a new capsular hepatic lesion. We introduced palliative chemotherapy with IV liposomal doxorubicin (40 mg/m^2^, every 4 weeks), which was stopped after two cycles, according to the patient’s wish, because of a grade 2 papular skin rash according to the CTC-AE definition. She refused further chemotherapy and was treated with palliative radiotherapy delivering 30 Gy in 5 fractions on the hepatic capsular lesion.

**Fig. 1:**
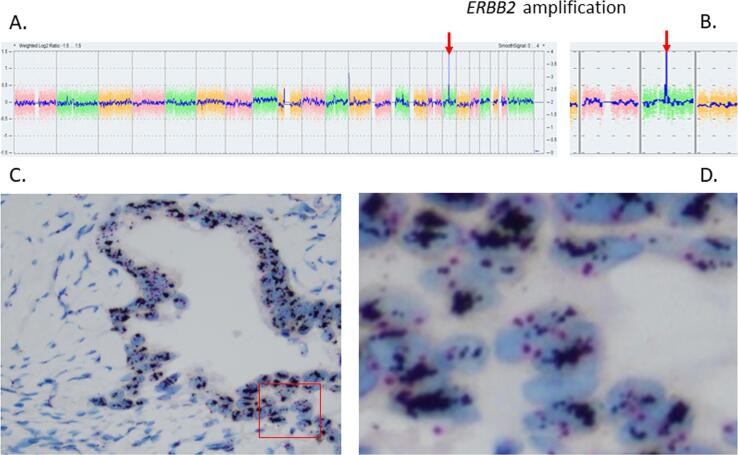
(A) Genomic profile generated by single-nucleotide polymorphism (SNP) array showing an amplification of *ERBB2* and absence of other significant copy number alterations. (B) Zoom on chromosome 17, showing the highly focal amplification involving *ERBB2.* (C-D) Representative HER2 chromogenic *in situ* hybridization (CISH) image showing the chromosome 17 amplification in all cancer cells. The HER2 (black)/CEP17 (red) ratio was visually estimated at 7.5.

Germline *BRCA1/BRCA2* testing performed soon after diagnosis did not reveal pathogenic variants. To explore further treatment opportunities, we performed a molecular analysis of the surgically resected tumor tissue, after the patient signed an informed consent. We did not find pathogenic variants in *BRCA1/BRCA2*, and next-generation sequencing with a 409-gene panel showed three variants of uncertain pathogenicity in *PRKDC, FLI1,* and *NTRK3* with a low allelic frequency (3–6%). None of these genes is known to be implicated in serous ovarian carcinoma (Bell et al., 2011). Copy number analysis showed a focal amplification of the 17q12 chromosomal region containing *ERBB*2, with at least 6 copies, which was confirmed by *in situ* hybridization (Fig. 1).

**Fig. 2:**
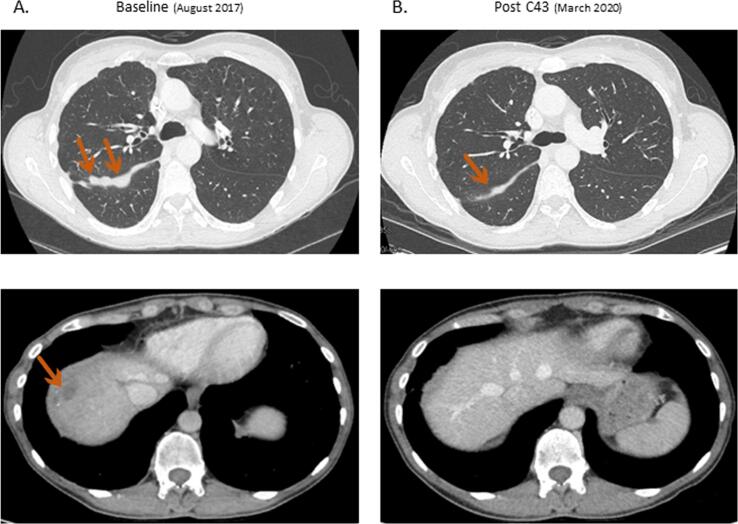
Contrast-enhanced computed tomography (CT) of the ovarian cancer lesions evolution. (A) At baseline, before starting the trastuzumab - pertuzumab therapy, right pleural thickening and one liver lesion. (B) After 43 trastuzumab – pertuzumab cycles (C), partial response with no liver lesion visible and right pleural thickness reduction.

Based on these results, a multidisciplinary molecular tumor board suggested treatment with trastuzumab (8 mg/kg for the first cycle and then 6 mg/kg, IV, every 3 weeks) and pertuzumab (840 mg for the first cycle and then 420 mg, IV, every 3 weeks), which we introduced in August 2017. The patient refused chemotherapy and only received the antibody combination. A baseline CT-scan before treatment introduction showed a slight increase in the pleural infiltration and a decrease in the capsular liver lesion after radiotherapy (Fig. 2A). Three months later, the first radiologic assessment showed a stable disease according to RECIST. At six months, the pleural infiltration and the hepatic lesion had decreased by 50%, fulfilling the criteria for a partial response.

Between the 9th and 12th months of trastuzumab-pertuzumab combination, and while maintaining it, she was treated with three cycles of pressurized intraperitoneal chemotherapy (PIPAC), because of the persistence of vague abdominal discomfort. So she received intraperitoneal cisplatin (5,5mg/m^2^) and doxorubicin (1.5 mg/m^2^) at another institution (CHUV, Switzerland), where a registry of patients treated with PIPAC is maintained (NCT03210298). The laparoscopic examination during the first PIPAC showed a peritoneal cancer index (PCI) score at 18, and the third and last laparoscopic exam a PCI score of 5, with a very good histologic response (peritoneal regression grading score (PRGS) 1–2), and negative cytology. The association of trastuzumab and pertuzumab has been maintained for over 37 months (52 cycles, August 2020) and has been clinically well tolerated, without any significant clinical or laboratory toxicity. The cardiac ultrasound, performed every quarter, remained normal throughout the treatment. After 13 months of treatment, measurable disease decreased by 85% from baseline, fulfilling the criteria for a partial response. In March 2020, after 32 months (43 cycles) of anti-HER2 combination, the CT scan showed a persistent partial response (Fig. 2B). Unfortunately, after 37 months of treatment, the disease progressed with only one new right apical pleural infiltrate, with others lesions still stable. After discussion in multidisciplinary tumor board, it has been decided to continue the trastuzumab-pertuzumab association with a radiotherapy targeting the new pulmonary lesion.

## 3 Discussion

The proto-oncogene *ERBB2* plays an essential role in coordinating the complex ErbB signaling network that regulates cell growth and differentiation. It can be amplified in 15–20% of primary breast cancer, and was synonymous with poor prognosis until the advent of HER2-directed therapies (Waks and Winer, 2019). Gastric and gastroesophageal cancers may also have *ERBB2* amplification in 4.4 to 53.4% of cases. In the advanced setting, trastuzumab associated with chemotherapy prolongs survival, but the addition of pertuzumab does not increase efficacy (Tabernero et al., 2018). More recently, HER2-directed therapy with trastuzumab and pertuzumab has been shown to be effective in colorectal cancer with *ERBB2* amplification (Meric-Bernstam et al., 2019). HER2 overexpression has been reported in several other cancer types, but the benefit from targeted therapies is variable and anecdotal, probably depending on the level of HER2 expression, the presence of tumor heterogeneity, and the state of activation of other pathways (Oh and Bang, 2019).

The efficacy of anti-HER2 agents is minimal in unselected patients with epithelial ovarian cancer (EOC). Most of the available studies are small and select patients based on varying HER2 expression criteria, or not at all. Different HER2-directed treatments, with or without chemotherapy, have been tested with little success. In phase II studies, pertuzumab monotherapy or in combination with gemcitabine, in molecularly unselected patients with advanced and recurrent EOC, only few responses were seen (Gordon et al., 2006, Makhija et al., 2010). In an another phase II study evaluating lapatinib with topotecan in EOC relapsing after first line platinum-therapy, there was no clear benefit (Lheureux et al., 2012).

Given the minimal response rate to anti-HER2 therapies in EOC, predicting which patients will benefit is hard. For example, the Gynecologic Oncology Group evaluated trastuzumab alone in a phase II study, with 41 patients having recurrent or refractory ovarian or primary peritoneal carcinoma with HER2 overexpression. Only 11.4% had a HER2 2 + or 3 + overexpression documented by immunohistochemistry, after screening 857 patients. Despite screening, the response rate was relatively low (7% achieving partial response), with a median PFS of 2 months (Bookman et al., 2003). In another study of seven patients progressing after one or two lines of standard treatment with confirmed HER2-positive status (FISH), three had a complete response and two had stable disease lasting three months with carboplatin, paclitaxel and trastuzumab combination (Guastalla et al., 2007).

In a more recent phase III trial, 156 patients with platinum-resistant HGSOC and low tumor *ERBB3* mRNA expression were treated with different chemotherapy backbones (topotecan, paclitaxel or gemcitabine), but no PFS or overall survival improvement was observed against placebo with the addition of pertuzumab (Lorusso et al., 2019). In a sub-protocol of the NCI-MATCH trial, trastuzumab emtansine (T-DM1) was given to three patients with ovarian cancer and resulted in stable disease of more than 6 months duration (Jhaveri et al., 2019).

We are not aware, to this date, of a study combining trastuzumab and pertuzumab in this context.

Our report argues in favor of the association of trastuzumab and pertuzumab in HGSOC with high-level focal amplification of *ERBB2*. We believe that the significant benefit observed in this case can be attributed to two factors. First, as in breast cancer, the combination of trastuzumab and pertuzumab might have greater efficacy than either drug alone and has not been previously tested in HGSOC. Second, the ovarian tumor of this patient demonstrated a very high-level focal amplification of *ERBB2* and no other notable driver gene mutations were found that could mediate resistance (Luque-Cabal et al., 2016). It is unclear whether *ERBB2* focal amplification is a better biomarker than HER2 overexpression, but it is generally present in a smaller number of patients and therefore more selective. In addition, the observed rate of HER2 overexpression in HGSOC has considerable variation in the literature, ranging from 8% to 66%, and noticeable intratumoral heterogeneity, which suggests low interobserver agreement and repeatability. We cannot estimate a specific copy-number cutoff for patients who are most likely to benefit, although our experience with this patient suggests that responses can occur with a HER2*/CEP17* ratio of 7 or more. In the NCI-MATCH trial (subprotocol Q) almost all patients who had more than 10 copies of *ERBB2* seemed to benefit from T-DM1 and both partial responses that were seen in that trial occurred in patients with more than 16 copies of *ERBB2* (Jhaveri et al., 2019)*.*

This case illustrates the subtleties of molecular tumor profiling. Based on our observation, further study of the trastuzumab and pertuzumab association is warranted in clinical trials with an appropriate exploration of companion biomarkers, especially the presence of focal *ERBB2* amplification and the absence of other driver gene alterations.

## 4 Materials and methods

Written informed consent was obtained from the patient for publication of this case report and accompanying images. A copy of the written consent is available for review by the Editor-in-Chief of this journal on request.

## CRediT authorship contribution statement

**Laure Thouvenin:** Conceptualization, Writing - original draft, Investigation. **Mélinda Charrier:** Writing - original draft, Writing - review & editing. **Sophie Clement:** Formal analysis, Writing - original draft, Writing - review & editing. **Yann Christinat:** Formal analysis, Writing - review & editing. **Jean-Christophe Tille:** Writing - review & editing. **Mauro Frigeri:** Writing - review & editing. **Krisztian Homicsko:** Writing - review & editing. **Olivier Michielin:** Writing - review & editing. **Alexandre Bodmer:** Writing - review & editing, Investigation. **Pierre O. Chappuis:** Writing - review & editing. **Thomas A. McKee:** Formal analysis, Resources, Writing - review & editing. **Petros Tsantoulis:** Writing - original draft, Writing - review & editing, Supervision.

## Declaration of Competing Interest

The authors declare that they have no known competing financial interests or personal relationships that could have appeared to influence the work reported in this paper.
